# Effectively Caring for Individuals With Behavioral and Psychological Symptoms of Dementia During the COVID-19 Pandemic

**DOI:** 10.3389/fpsyt.2020.573367

**Published:** 2020-10-06

**Authors:** Alvin Keng, Eric E. Brown, Aviva Rostas, Tarek K. Rajji, Bruce G. Pollock, Benoit H. Mulsant, Sanjeev Kumar

**Affiliations:** ^1^ Adult Neurodevelopmental and Geriatric Psychiatry Division, Centre for Addiction and Mental Health, Toronto, ON, Canada; ^2^ Department of Psychiatry, University of Toronto, Toronto, ON, Canada

**Keywords:** Alzheimer’s disease and related disorders, behavioral and psychological symptoms of dementia, COVID-19, pandemic, coronavirus, clinical care, clinical research, caregivers

## Abstract

The COVID-19 pandemic has significantly affected the elderly and particularly individuals with Alzheimer’s disease and related disorders (ADRD). Behavioral and psychological symptoms of dementia (BPSD) are heterogeneous and common in individuals with ADRD and are associated with more severe illness. However, unlike the cognitive symptoms of ADRD that are usually progressive, BPSD may be treatable. Individuals with BPSD are facing unique challenges during the pandemic due to the inherent nature of the illness and the biological and psychosocial impacts of COVID-19. These challenges include a higher risk of severe COVID-19 infection in individuals with BPSD due to their frailty and medical vulnerability, difficulty participating in screening or testing, and adhering to infection control measures such as physical distancing. Further, biological effects of COVID-19 on the brain and its psychosocial impact such as isolation and disruption in mental health care are likely to worsen BPSD. In this paper, we discuss these challenges and strategies to manage the impact of COVID-19 and to effectively care for individuals with BPSD in community, long-term care, or hospital settings during the pandemic. Despite the ongoing uncertainty associated with this pandemic, we can reduce its impact on individuals with BPSD with a proactive approach.

## Introduction

The COVID-19 pandemic has affected the elderly including those with Alzheimer’s disease and related disorders (ADRD), creating numerous challenges to their mental health (1, 2). Behavioral and psychological symptoms of dementia (BPSD) affect the majority of individuals with ADRD (3). BPSD are a group of heterogeneous symptoms that include motor disturbances, disinhibition, hyperactivity, psychosis, euphoria, affective symptoms, apathy, eating disturbances, and night-time behaviors (3, 4). BPSD occur at all stages of cognitive disorders including pre-clinical, mild cognitive impairment, or dementia (5). Furthermore, specific cognitive disorders may present with different BPSD (6–8). BPSD are associated with more rapid cognitive decline and poor functional status (9, 10). BPSD are widely prevalent in residents of long-term care homes (11, 12) where the current pandemic has had the most devastating effect (13). Acutely, BPSD may require emergency room assessment and hospital admission (14), potentially exposing patients to nosocomial COVID-19.

Older age, medical comorbidities, and other risk factors, such as APOE4 (15), which are commonly seen in individuals with BPSD, are also associated with increased risk of severe COVID-19 infection and mortality (16–18). Further, it has been shown that up to 69% of patients with severe COVID-19 infection may present with delirium or encephalopathy (19), which increase mortality rates (20). In the United States, the case fatality rate for those ≥85 years old has been reported to be between 10 and 27%, about 100-fold higher than the rate for those 20–44 years old (18). These studies did not report separately on the subgroups with dementia or BPSD; however up to 40% of elderly ≥85 years old are likely to have ADRD with associated BPSD in a significant proportion (21). Given the association of BPSD with risk factors of both COVID-19 exposure and severity, we expect that those with BPSD are one of the most vulnerable groups and that the pandemic will make their care more challenging.

The recommended treatment approach to BPSD depends on the presenting symptom or the nature of the underlying disorder. However, individualized non-pharmacologic interventions are typically first line, followed by carefully considered pharmacological interventions (22, 23). Furthermore, optimal management of BPSD requires a multidisciplinary collaborative approach between physicians, allied health clinicians, behavioral therapists, and patients’ substitute decision makers (24). Standard interventions for BPSD involve close contact between patients and their caregivers (3, 24). During the pandemic, these interventions may require significant adaptation or restriction to be compatible with measures to reduce infection risk including “physical distancing” (25) or “social distancing” (26).

In a 4-year retrospective case-control study of an Alzheimer’s Special Care Unit, a higher inherent risk of respiratory infections relative to other units was found (27). Previous experiences of infectious disease outbreaks offer some lessons to balance effective management of BPSD with infection control principles (28–30). However, these interventions are limited in scope and do not capture the unprecedented scale of the current pandemic.

There is a need to understand the impact of the current pandemic on individuals with BPSD across various settings from community living to hospital units. Further, there is an urgent need to implement preventive interventions to protect individuals with ADRD from the COVID-19 infection while effectively managing BPSD. In this paper, we discuss unique challenges faced by individuals with BPSD and their caregivers during the COVID-19 pandemic and provide recommendations on how to address these challenges. Our aim is to address these challenges in individuals experiencing significant BPSD across the spectrum of cognitive decline ranging from pre-clinical to dementia, and across different neurodegenerative disorders.

## Higher Risk of COVID-19 Infection and Associated Morbidity in Individuals With BPSD

### Risk of COVID-19 Infection and Its Severity

**Figure 1:**
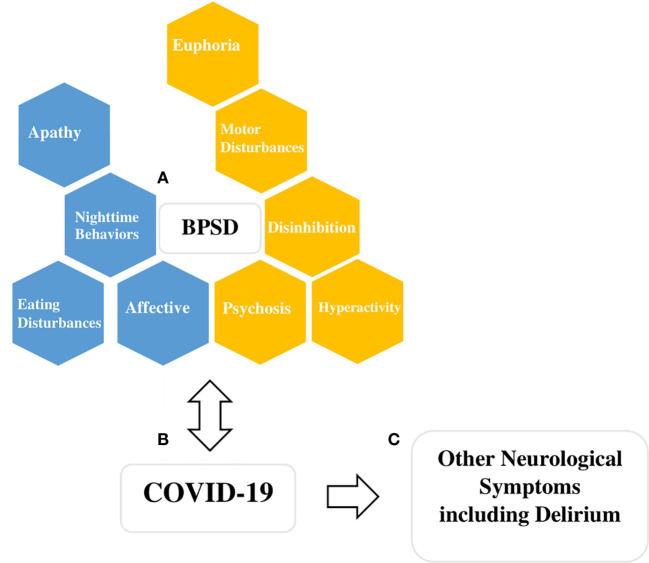
Figure describing potential interactions between COVID-19 and behavioral and psychological symptoms of dementia (BPSD). **(A)** BPSD are clustered here, based on previous consensus, into four main groups (hyperactivity, affective, psychosis, euphoria), and five other symptoms are listed individually (disinhibition, motor disturbances, apathy, night-time behaviors, eating disturbances) (4). Boxes colored gold indicate BPSD symptoms or clusters that may get worse due to the biological or psychological impact of COVID-19, and also the symptoms themselves interfere with infection control precautions and thus increase the chances of spread of COVID-19 infections (*i.e.* individuals with an increased propensity to wander or decreased likelihood of cooperating with isolation). Boxes colored blue indicate symptoms or clusters that are likely to worsen due to the biological or psychosocial impact of the COVID-19 but may not present challenges from infection control perspective (*i.e.* social isolation, loss of scheduled activities and routines). **(B)** COVID-19 and its hypothetical bidirectional relationship with BPSD, emphasizing the risk of more severe COVID-19 infection in individuals with BPSD due to their frailty and medical vulnerability. **(C)** COVID-19 can present with neurological symptoms and delirium due to its biological impact on the brain and nervous system and other systemic effects. Delirium and other neurological symptoms may also mimic BPSD.

There is increasing interest in the possible association between BPSD and COVID-19 infection (31, 32) and the challenge this may pose for those who care for individuals with BPSD (33). Although the association between BPSD and COVID-19 risk and severity is yet to be established empirically, the literature on this topic is expanding quickly. First, individuals with BPSD experiencing motor disturbances, disinhibition, hyperactivity, and psychosis may place themselves at higher risk of infection by increasing their proximity to others (**Figure 1**). Second, BPSD are associated with increased severity of cognitive impairment which limits the individual’s ability to understand, remember, and therefore, adhere to instructions regarding isolation or hand hygiene (9). Third, BPSD are associated with anosognosia, limiting the individual’s ability to adjust their behaviors, take necessary precautions, and seek help (34). This poor insight has been shown to increase their care needs and use of support services, which are critical resources during this pandemic (35). Fourth, individuals with BPSD and severe cognitive impairment depend on others for their basic needs which may involve close physical contact and potential exposure to a range of situations including personal care, feeding, and behavioral support for complex activities of daily living (36). Fifth, most health and personal care workers serve many patients or several facilities thus increasing the risk of infection. Sixth, environmental factors, such as shared rooms and physical layout, may limit an individual’s ability to isolate. Finally, dementia is associated with frailty, a syndrome of physical symptoms (*i.e.* weight loss, exhaustion, weakness, and inactivity) and functional decline and dysregulation of immune and inflammatory mechanisms (37, 38). This places patients with dementia at a higher risk of infection and mortality when exposed to the virus. Individuals with BPSD are likely to experience even more frailty among those with dementia due to the nature of their symptoms (39).

BPSD such as apathy, affective symptoms, and psychosis may impact the individuals’ ability to report symptoms of the infection. Older age has been identified as a risk factor for mortality from COVID-19 infection (17, 18). Furthermore, emerging evidence suggests an association between APOE4, a specific risk factor for Alzheimer’s Disease, and the risk and severity of infection (15, 40). Dementia has also been reported as a common comorbidity (12%) in those who have died due to COVID-19 (41) even though it may be under-represented in studies of in-hospital deaths as individuals with severe dementia may not be transferred to hospital. Individuals with BPSD also have comorbidities that result in a poorer prognosis when they are hospitalized (42, 43). Moreover, medications used in the management of BPSD, such as benzodiazepines and antipsychotics, may increase cardiovascular and respiratory mortality through sedation, cardiac toxicity, or increased risk of aspiration (44). To summarize, the risk of infection and its severity seems to be elevated in individuals with BPSD and their caregivers. Special consideration should be given to individuals with BPSD when planning preventive and therapeutic initiatives for COVID-19, keeping in mind the unique vulnerabilities of this population.

### Screening and Testing for COVID-19 in Individuals With BPSD

Specific BPSD such as apathy, affective symptoms, and psychosis, as well as moderate or severe cognitive impairment may result in inadequate participation in screening questionnaires and testing for COVID-19. Consequently, the task of monitoring and screening will fall on family and professional caregivers in the community, or nursing and other allied healthcare workers (HCW) in long-term care homes and inpatient units. Caregivers and HCW need to assess for both typical (upper respiratory symptoms and fever) and atypical presentations of COVID-19, including gastrointestinal and neurological symptoms (17, 45). An acute change in behavior and delirium might be the first manifestation of an underlying infection (20). Hyperactive presentations of delirium may interfere with COVID-19 screening. Clinicians and organizations should employ structured algorithms and routinely include COVID-19 screening in delirium work-up of individuals with BPSD (46). Unfortunately, nasopharyngeal swabs for COVID-19 are invasive and require patient cooperation to obtain an adequate specimen (47, 48). This may pose a challenge in certain individuals with BPSD. Individuals with BPSD who experience agitation/hyperactivity, or disinhibition may have higher false negative rates due to poor compliance when compared to those with apathy or affective symptoms. Thus, continued universal precautions for infection control and aggressive testing may be necessary. Individuals with BPSD living in the community may be unable to access screening and testing for COVID-19 and require support from agencies and primary care providers.

### Infection Control Precautions for Individuals With BPSD During COVID-19

Adherence to infection control precautions may be impeded by BPSD (49, 50). Specifically symptoms such as motor disturbances, euphoria, disinhibition, hyperactivity, and psychosis may impair the patients’ ability to maintain isolation, stay in one place or wear face masks (**Figure 1**). In such cases, use of behavioral and pharmacological interventions may need to be optimized. It is still important to use the least restrictive measures specific to each situation, such as creating physical barriers or cues by rearranging furniture or changing the layout of common areas to prevent wandering and to restrict movement of individuals to certain areas. In some cases, upholding infection control principles may require the use of chemical restraints (*e.g.* sedative medications in an emergency situation to reduce movement), as well as seclusion, or physical restraints as a last resort. However, these situations require careful examination of ethical, legal, and institutional factors, due to the potential for serious harm (51).

### Communicating COVID-19 Risks to Individuals With BPSD and Their Caregivers

Individuals with BPSD may have varying levels of cognitive impairment. Those with more severe cognitive impairment may be unable to appreciate the risks and consequences of the illness for themselves and others. Individuals with milder or non-amnestic cognitive impairment may have some preserved ability to understand and practice basic infection control measures. Nevertheless, communicating the risks of infection is critical to elicit cooperation with infection control measures. Verbal and non-verbal modes of communication should be used, moving from basic to more complicated principles of infection control. Non-verbal measures that have been studied to improve communication between individuals with ADRD and staff include: memory books, visual and motor cues, multi-sensory stimulation Snoezelen interventions, and active listening techniques (*e.g.* making eye contact) (52). The communication needs to be individualized based on personal and environmental factors (50, 53, 54). For example, an individual with psychosis or severe cognitive impairment may not fully comprehend the pandemic but may be directed to wash their hands with frequent reminders. Family caregivers may be reluctant to share information regarding infection risk for fear of aggravating symptoms and should be encouraged and supported. Caregiver based interventions are highly effective for management of BPSD and can help with reducing caregiver stress (55). Several organizations have published helpful resources and run support groups specific to COVID-19 (53, 54). Further, due to shortage of resources to care for patients with COVID-19 infection, individuals with ADRD or BPSD may be triaged to a lower priority, as has occurred in some jurisdictions, sparking ethical considerations (56, 57). The substitute decision makers of individuals with ADRD and BPSD should be involved in these discussions to promote informed choices (2, 58). The presence of BPSD may influence them and lead them to select a palliative approach, without realizing that BPSD is usually treatable and temporary (3, 22).

## Managing the Biological and Psychosocial Impact of COVID-19 on BPSD

### Biological Impact of COVID-19 on BPSD and Considerations for Appropriate Use of Psychopharmacology

Mounting evidence suggests that COVID-19 causes possible neuronal death *via* neuro-inflammatory mechanisms or vascular mechanisms such as hyper-coagulation (59, 60). Early studies from Wuhan, China reported that within days of admission, over 1/3 of patients with COVID-19 had one or more neurologic symptoms (*i.e.* dizziness, headache, impaired consciousness) (45). These findings are now supported in other cohorts (61) with neuropathological (62) and MRI correlates (63). In severe cases, COVID-19 patients are at higher risk of stroke, delirium, and acute encephalopathy, leading to both short and long-term neuropsychiatric sequelae (64, 65) and causing significant problems with management in hospital and ICU settings (66, 67). Individuals with ADRD are particularly vulnerable to neuropsychiatric impact of any systemic illness and are likely to experience even higher rates of delirium and encephalopathy, which can be mistaken for BPSD (20). Increasingly, healthcare organizations and public health entities are including these symptoms in screening algorithms (25). However, given the focus on the respiratory illness associated with COVID-19, its neuropsychiatric manifestations are likely be missed or to be mistaken for pre-existing BPSD. Clinicians should consider new acute neuropsychiatric symptoms or worsening in BPSD to be an indication for COVID-19 testing. Long term neurologic sequelae could also be linked to COVID-19 infection due to neurodegenerative changes associated directly with the virus or indirectly with autoimmune processes. These sequelae could mimic some neurodegenerative syndromes, warranting long-term follow up (68).

Some individuals who experience worsening of BPSD due to COVID-19 may require additional pharmacological interventions. Many of these individuals are already prescribed multiple psychiatric medications and are likely to experience adverse effects related to polypharmacy (69, 70). This situation may worsen further due to lack of access to specialist care, limited resources, and a desire for faster symptom relief in the context of COVID-19 (2). Clinicians should adhere to best practice guidelines: first optimizing non-pharmacological measures, then carefully weighing benefits and risks of pharmacological interventions (71). Algorithms or integrated care pathways may help in treatment planning (23). We suggest a careful review of current medications and considering discontinuation of ineffective medications or those with potential for drug interactions or adverse effects, followed by a sequential trials of safer evidence-based medications (23). Special consideration should be given to the use of benzodiazepines and other sedating medications in concurrent BPSD and COVID-19 given the risk of respiratory depression (72). Similarly, COVID-19 has known cardiac complications including heart failure and arrhythmias (73). Based on this information and in keeping with general principles of treatment in geriatric medicine, we suggest avoiding or exercising extra caution with medications that prolong QTc or have other cardiac adverse effects (74). We advocate for use of an individualized algorithmic approach to pharmacological management of BPSD in each patient with emphasis on monotherapy, measurement based care, and close monitoring for cardiac and other potential adverse effects (23).

### Psychosocial Impact of COVID-19 on BPSD and Its Management

We expect an increase in all domains of BPSD (**Figure 1**) in keeping with projected worsening of pre-existing mental health symptoms in the general population (19, 75, 76). First, cancellation of recreational activities and routine disruption are particularly challenging for individuals with BPSD. Second, physical distancing and infection control measures may result in a reduction of visits from family, friends, and caregivers leading to increased social isolation and worsening of affective symptoms, such as anxiety and depressed mood (50, 77). Third, individuals with BPSD who are able to comprehend some aspects of the pandemic may also experience second-hand distress from caregivers (50). Lastly, individuals with BPSD may find it harder to adequately use telecommunications and virtual care tools that may help them cope with the psychosocial impact of the pandemic. As individuals with BPSD live in a variety of settings, we discuss specific measures that can be adapted at each setting.

#### Home or Community Living

In home environments, family or external caregivers provide support for activities of daily living and management of BPSD. Appointments with physicians and other clinicians may have to be conducted virtually (2). Thus, family caregivers and clinicians should develop an inventory of existing supports for the individual during the pandemic. The goal should be to continue to treat individuals with BPSD at home, where the risk of exposure to COVID-19 is lower, by ensuring that healthy home routines are continued, and unmet needs are identified and addressed (2). Individuals with BPSD and their caregivers should be engaged in discussions regarding protocols for minimizing exposure to COVID-19 during in-person visits. Goals of care and a plan for transfer to primary, secondary, or tertiary care centers should be discussed explicitly with the individuals and their substitute decision makers as applicable.

#### Long-Term Care Homes (LTCH)

LTCH have been a major focus during this pandemic, given the high morbidity and mortality in these settings (78, 79). Many LTCH face challenges in terms of staff absenteeism due to COVID-19 morbidity, daycare/school closures, or rules preventing staff from working in more than one health facilities. Behavioral support teams and specialist care clinics may not be functioning at their optimal level (80). Furthermore, many LTCH have invoked blanket bans on visitors to their facilities. Although these measures were implemented to protect residents, there is now evidence that such measures lead to increased social isolation and worsening of depression and anxiety (81). As much as possible, LTCH should preserve some programming to prevent decompensation while following universal precautions. For example, audio-video phone conferencing, physical exercises, music, doll therapy, and individualized one-to-one relaxation training can be safely used in residents’ personal space (82). When LTCH residents with BPSD experience death of peer residents due to COVID-19, some may benefit from grief counseling or supportive therapy (83). LTCH should also revisit advance directives with residents and their substitute decision makers in view of the pandemic. Residents’ wishes regarding code status, transfer to hospital or ICU, and provision of invasive care should be ascertained (84).

#### Hospital and Other Behavioral Units

At any given time, a significant number of patients affected by dementia and BPSD are admitted to specialized behavioral units or geriatric inpatient units (14, 85). There may be inadequate behavioral and psychosocial interventions due to staff unavailability or diversion towards infection control activities. Many hospitals limit group activities due to infection control. Thus, they need to maximize the individualized one-to-one behavioral interventions either in person or through audio-video technologies, which might in-fact require more staff resources (86). To meet these demands, hospitals may need to redeploy staff from other clinical services such as outpatient clinics, or other services deemed “non-essential”. Volunteers may also be able to provide psychosocial support to older inpatients when their engagement is allowed by local policies and directives (87).

### Attending to the Needs of Those Working and Caring for Individuals With BPSD

Consistent staffing is critical to provide effective care to individuals with BPSD because the work demands a high degree of familiarity with the individual. The psychological impact of working in LTCH, hospital, or other institutional settings during a pandemic should be recognized and addressed proactively (88). There are many potential sources of stress for HCW including caring for vulnerable and potentially dying residents, keeping abreast of regularly evolving infection control regulations, and worrying about their own health and safety. Frontline HCW involved in the care of patients with COVID-19 have higher risks of mental distress, insomnia, anxiety, and depression (89). Frontline HCW should have support made available, but not mandated (90). Organizations should have clear and widely advertised ways for staff to access timely and confidential professional support and crisis services. Including mental health professionals in planning and supporting teams may be helpful. The overall resilience of HCW raises the hope that the healthcare work force can be preserved with adequate measures (91). Non-HCW, such as family, friends, and informal caregivers, play a critical role in the care of individuals with BPSD and might be experiencing stress due to reduction in the frequency of family visits during the pandemic (92, 93). Thus, efforts should be made to proactively detect and manage caregiver stress among family members and other informal caregivers (93).

## Conclusions

The COVID-19 pandemic is disproportionately impacting the elderly including those with BPSD. Individuals with BPSD and their family or professional caregivers are facing unique challenges due to the inherent nature of the illness and superimposed biological and psychosocial factors related to the COVID-19 pandemic. Certain BPSD may lead to a higher risk of infection, a more severe course of illness, and higher mortality rates. These challenges can be addressed with a proactive approach. It is important to implement infection control strategies for individuals with BPSD across settings such as proactive screening and testing, maintaining a high degree of suspicion for atypical presentations of COVID-19, and instituting timely interventions. Individuals with BPSD and COVID-19 should also be monitored for long term biological and psychosocial effects of COVID-19. BPSD need to be managed during the pandemic using evidence-based structured psychosocial and biological interventions through innovative means such as virtual and individualized care, use of structured and algorithmic models of care, and appropriate use of psychosocial interventions across healthcare settings. Individuals with BPSD and their substitute decision makers should be invited to discuss and make decisions regarding goals of care and end of life care. Efforts should be made to address the psychological health of the frontline HCW and informal caregivers as they are paramount to success in caring for BPSD.

## Data Availability Statement

The original contributions presented in the study are included in the article/supplementary material; further inquiries can be directed to the corresponding author.

## Author Contributions

AK: Substantial contributions to the conception or design of the work and the acquisition, analysis, or interpretation of data for the work; drafting the work or revising it critically for important intellectual content; final approval of the version to be published; and agreement to be accountable for all aspects of the work in ensuring that questions related to the accuracy or integrity of any part of the work are appropriately investigated and resolved. EB: Substantial contributions to the conception or design of the work; or the acquisition, analysis, or interpretation of data for the work; and final approval of the version to be published. AR: Substantial contributions to the conception or design of the work; or the acquisition, analysis, or interpretation of data for the work; and final approval of the version to be published. TR: Substantial contributions to the conception or design of the work; or the acquisition, analysis, or interpretation of data for the work; and final approval of the version to be published. BP: Substantial contributions to the conception or design of the work; or the acquisition, analysis, or interpretation of data for the work; and final approval of the version to be published. BM: Substantial contributions to the conception or design of the work; interpretation of data for the work; revising it critically for important intellectual content; and final approval of the version to be published. SK: Substantial contributions to the conception or design of the work and the acquisition, analysis, or interpretation of data for the work; drafting the work or revising it critically for important intellectual content; final approval of the version to be published; and agreement to be accountable for all aspects of the work in ensuring that questions related to the accuracy or integrity of any part of the work are appropriately investigated and resolved. All authors contributed to the article and approved the submitted version.

## Conflict of Interest

TR: research support from Brain Canada, Brain and Behavior Research Foundation, BrightFocus Foundation, Canada Foundation for Innovation, Canada Research Chair, Canadian Institutes of Health Research, Centre for Aging and Brain Health Innovation, National Institutes of Health, Ontario Ministry of Health and Long-Term Care, Ontario Ministry of Research and Innovation, and the Weston Brain Institute. He also received in-kind equipment support for an investigator-initiated study from Magstim, and in-kind research accounts from Scientific Brain Training Pro. BP: research support from the Peter & Shelagh Godsoe Endowed Chair in Late-Life Mental Health, CAMH Foundation and Discovery Fund, National Institute of Aging, Brain Canada, the Canadian Institutes of Health Research, the Alzheimer’s Drug Discovery Foundation, the Ontario Brain Institute, the Centre for Aging and Brain Health Innovation, the Bright Focus Foundation, the Alzheimer’s Society of Canada, the W. Garfield Weston Foundation, the Weston Brain Institute, the Canadian Consortium on Neurodegeneration in Aging and Genome Canada. Honoraria from the American Geriatrics Society. He holds United States Provisional Patent No.62/466,651 for a cell-based assay and kits for assessing serum anticholinergic activity. BM: research financial support from Brain Canada, CAMH Foundation, Canadian Institutes for Health Research, and US National Institutes of Health; nonfinancial support from Pfizer (medication for an NIH-funded trial), Eli Lilly (medication and matching placebo for an NIH-funded trial), Capital Solution Design LLC (software for a trial funded by the CAMH Foundation), and HAPPYneuron (software for a trial funded by Brain Canada). He directly owns shares of General Electric (less than $5,000). SK: research support from Brain and Behavior Foundation, National institute on Ageing, BrightFocus Foundation, Brain Canada, Canadian Institute of Health Research, Centre for Ageing and Brain Health Innovation, Centre for Addiction and Mental Health, University of Toronto. He also receives equipment support from Soterix Medical.

The remaining authors declare that the research was conducted in the absence of any commercial or financial relationships that could be construed as a potential conflict of interest.

## References

[1] Kar SK Yasir Arafat SM Kabir R Sharma P Saxena SK Coping with Mental Health Challenges During COVID-19. In: Saxena SK, editor. Coronavirus Disease 2019 (COVID-19): Epidemiology, Pathogenesis, Diagnosis, and Therapeutics. Singapore: Springer Singapore (2020), p. 199–213. 10.1007/978-981-15-4814-7_16

[2] Brown EE Sc M Kumar S Anticipating and Mitigating the Impact of the COVID-19 Pandemic on Alzheimer’s Disease and Related. Am J Geriatr Psychiatry (2020) 7:712–21. 10.1016/j.jagp.2020.04.010 PMC7165101 32331845

[3] Bessey LJ Walaszek A Management of Behavioral and Psychological Symptoms of Dementia. Curr Psychiatry Rep (2019) 21:1–11. 10.1007/s11920-019-1049-5 31264056

[4] van der Linde RM Dening T Matthews FE Brayne C Grouping of behavioural and psychological symptoms of dementia. Int J Geriatr Psychiatry (2014) 29:562–8. 10.1002/gps.4037 PMC4255309 24677112

[5] Scaricamazza E Colonna I Sancesario GM Assogna F Orfei MD Franchini F Neuropsychiatric symptoms differently affect mild cognitive impairment and Alzheimer’s disease patients: a retrospective observational study. Neurol Sci (2019) 40:1377–82. 10.1007/s10072-019-03840-4 30903419

[6] Spalletta G Long JD Robinson RG Trequattrini A Longitudinal Neuropsychiatric Predictors of Death in Alzheimer ‘ s Disease. J Alzheimer’s Dis (2015) 48:627–36. 10.3233/JAD-150391 26402103

[7] Fernández-Matarrubia M Matías-Guiu JA Cabrera-Martín MN Moreno-Ramos T Different apathy clinical pro fi le and neural correlates in behavioral variant frontotemporal dementia and Alzheimer’s disease. Int J Geriatr Psychiatry (2018) 33:141–50. 10.1002/gps.4695 28240379

[8] Fernández-Matarrubia M Cabrera-Martín MN Moreno-Ramos T Matías-Guiu JA Behavioural variant frontotemporal dementia: Clinical and therapeutic approaches. Neurol English Ed (2014) 29:464–72. 10.1016/j.nrleng.2013.03.004 23648383

[9] van der Linde RM Dening T Stephan BCM Prina AM Evans E Brayne C Longitudinal course of behavioural and psychological symptoms of dementia: systematic review. Br J Psychiatry (2016) 209:366–77. 10.1192/bjp.bp.114.148403 PMC5100633 27491532

[10] Ismail Z Smith EE Geda Y Sultzer D Brodaty H Smith G Neuropsychiatric symptoms as early manifestations of emergent dementia : Provisional diagnostic criteria for mild behavioral impairment. Alzheimer's & Dementia (2016) 12:195–202. 10.1016/j.jalz.2015.05.017 PMC4684483 26096665

[11] Seitz D Purandare N Conn D Prevalence of psychiatric disorders among older adults in long-term care homes: A systematic review. Int Psychogeriatrics (2010) 22:1025–39. 10.1017/S1041610210000608 20522279

[12] Arai A Ozaki T Asuna K Behavioral and psychological symptoms of dementia in older residents in long-term care facilities in Japan: A cross-sectional study. Aging Ment Health (2017) 21:1099–105. 10.1080/13607863.2016.1199013 27333434

[13] D’Adamo H Yoshikawa T Ouslander JG Coronavirus Disease 2019 in Geriatrics and Long-Term Care: The ABCDs of COVID-19. J Am Geriatr Soc (2020) 68:912–17. 10.1111/jgs.16445 32212386

[14] Canadian Institute for Health Information Dementia in Hospitals (2020). Available at: https://www.cihi.ca/en/dementia-in-canada/dementia-across-the-health-system/dementia-in-hospitals (Accessed June 2, 2020).

[15] Goldstein MR Poland GA Graeber CW Does apolipoprotein E genotype predict COVID-19 severity? QJM (2020) 113:529–30. 10.1093/qjmed/hcaa142 PMC7197574 32339247

[16] Onder G Rezza G Brusaferro S Case-Fatality Rate and Characteristics of Patients Dying in Relation to COVID-19 in Italy. JAMA J Am Med Assoc (2020) 2019:2019–20. 10.1001/jama.2020.4683 32203977

[17] Zhou F Yu T Du R Fan G Liu Y Liu Z Clinical course and risk factors for mortality of adult inpatients with COVID-19 in Wuhan, China : a retrospective cohort study. Lancet (2020) 395:1054–62. 10.1016/S0140-6736(20)30566-3 PMC7270627 32171076

[18] Bialek S Boundy E Bowen V Chow N Cohn A Dowling N Severe outcomes among patients with coronavirus disease 2019 (COVID-19) - United States, February 12-march 16, 2020. Morb Mortal Wkly Rep (2020) 69:343–6. 10.15585/mmwr.mm6912e2 PMC7725513 32214079

[19] Rogers JP Chesney E Oliver D Pollak TA Mcguire P Fusar-Poli P Psychiatric and neuropsychiatric presentations associated with severe coronavirus infections: a systematic review and meta-analysis with comparison to the COVID-19 pandemic. Lancet Psychiatry (2020) 7 (7):611–27. 10.1016/S2215-0366(20)30203-0 PMC7234781 32437679

[20] Inouye SK Delirium in older persons. N Engl J Med (2006) 354:2510. 10.1056/NEJMra052321 16540616

[21] Alzheimer’s Association Report 2020 Alzheimer’s disease facts and figures. Alzheimer’s Dement (2020) 16:391–460. 10.1002/alz.12068

[22] Kales HC Gitlin LN Lyketsos CG Assessment and management of behavioral and psychological symptoms of dementia. BMJ (2015) 350:1–16. 10.1136/bmj.h369 PMC4707529 25731881

[23] Davies SJC Burhan AM Kim D Gerretsen P Graff-Guerrero A Woo VL Sequential drug treatment algorithm for agitation and aggression in Alzheimer’s and mixed dementia. J Psychopharmacol (2018) 32:509–23. 10.1177/0269881117744996 PMC5944080 29338602

[24] Caspar S Macdonald SWS Clinical features and multidisciplinary approaches to dementia care. J Multidiscip Healthc (2011) 2011(4):125–47. 10.2147/JMDH.S17773 PMC3104685 21655340

[25] World Health Organization COVID-19 Strategy Update (2020). Available at: https://www.who.int/publications/i/item/covid-19-strategy-update—14-april-2020 (Accessed June 2, 2020).

[26] Social Distancing, Quarantine, and Infection Control (Centers for Disease Control and Prevention) (2020). Available at: https://www.cdc.gov/coronavirus/2019-ncov/prevent-getting-sick/social-distancing.html (Accessed May 20, 2020).

[27] Perls TT Herget M Higher Respiratory Infection Rates on an Alzheimer ‘ s Special Care Unit and Successful Intervention. J Am Geriatr Soc (1995) 43(12):1341–4. 10.1111/j.1532-5415.1995.tb06611.x 7490383

[28] Grota PG Investigating an Outbreak of Conjunctivitis in a 46 Bed Veterans Administration Dementia Unit. Am J Infect Control (2007) 35(5):E181. 10.1016/j.ajic.2007.04.264

[29] Osbourn M McPhie KA Ratnamohan M Dwyer DE Durrheim D Outbreak of human metapneumovirus infection in a residential aged care facility. Comm Dis Intell Q Rep 33 1 (2009):39–41. 10.33321/cdi.2009.33.8 19618769

[30] Honda H Iwahashi J Kashiwagi T Imamura Y Hamada N Anraku T Letter to Editor: Outbreak of Human Metapneumovirus in Elderly Inpatients in Japan. J Am Geriatr Soc (2006) 54:177–80. 10.1111/j.1532-5415.2005.00575_10.x PMC7167045 16420227

[31] Devita M Bordignon A Sergi G Coin A The psychological and cognitive impact of Covid-19 on individuals with neurocognitive impairments: research topics and remote intervention proposals. Aging Clin Exp Res (2020), 1–4. 10.1007/s40520-020-01637-6 32583375 PMC7314284

[32] Lennon JC Neurologic and Immunologic Complications of COVID-19: Potential Long-Term Risk Factors for Alzheimer’s Disease. J Alzheimer’s Dis Rep (2020) 4:217–21. 10.3233/adr-200190 PMC7369139 32715280

[33] Martin-Khan M Bail K Graham F Thompson J Yates MW Cognitive Impairment and COVID-19 Hospital Care Guidance Committee. Interim guidance for the care of adult patients with cognitive impairment requiring hospital care during the COVID-19 pandemic in Australia. University of Queensland (2020). Available at: https://chsr.centre.uq.edu.au/interim-guid-ance-care-adult-patients-cognitive-impairment-requir-ing-hospital-care-during-covid-19-pandemic-australia.

[34] Spalletta G Girardi P Caltagirone C Orfei MD Anosognosia and neuropsychiatric symptoms and disorders in mild alzheimer disease and mild cognitive impairment. J Alzheimer’s Dis (2012) 29:761–72. 10.3233/JAD-2012-111886 22349686

[35] Turró-Garriga O Garre-Olmo J Reñé-Ramírez R Calvó-Perxas L Gascón-Bayarri J Conde-Sala JL Consequences of Anosognosia on the Cost of Caregivers’ Care in Alzheimer’s Disease. J Alzheimer’s Dis (2016) 54:1551–60. 10.3233/JAD-160419 27636844

[36] Beeri MS Werner P Davidson M Noy S The cost of behavioral and psychological symptoms of dementia (BPSD) in community dwelling Alzheimer’s disease patients. Int J Geriatr Psychiatry (2002) 17:403–8. 10.1002/gps.490 11994927

[37] Kulmala J Nykänen I Mänty M Hartikainen S Association between frailty and dementia: A population-based study. Gerontology (2013) 60:16–21. 10.1159/000353859 23970189

[38] Li H Manwani B Leng SX Frailty, inflammation, and immunity. Aging Dis (2011) 2:466–73. PMC3295062 22396895

[39] Sugimoto T Ono R Kimura A Saji N Niida S Toba K Physical Frailty Correlates With Behavioral and Psychological Symptoms of Dementia and Caregiver Burden in Alzheimer’s Disease. J Clin Psychiatry (2018) 79:e1–7. 10.4088/JCP.17m11991 30474939

[40] Kuo C-L Pilling LC Atkins JL Masoli JA Joao D Kuchel GA APOE E4 Genotype Predicts Severe COVID-19 in the UK Biobank Community Cohort. medRxiv (2020) 1–7. 10.1101/2020.05.07.20094409 PMC7314139 32451547

[41] Cipriani G Di Fiornio M Letter to Editor: Access to Care for Dementia patients suffering from COVID-19. Am J Geriatr Psychiatry (2020) 28 (7):796–97. 10.1016/j.jagp.2020.04.009 PMC7162751 32327300

[42] Kunik ME Snow AL Molinari VA Menke TJ Souchek J Sullivan G Health care utilization in dementia patients with psychiatric comorbidity. Gerontologist (2003) 43:86–91. 10.1093/geront/43.1.86 12604749

[43] Martín-García S Rodríguez-Blázquez C Martínez-López I Martínez-Martín P Forjaz MJ Comorbidity, health status, and quality of life in institutionalized older people with and without dementia. Int Psychogeriatrics (2013) 25:1077–84. 10.1017/S1041610213000458 23575107

[44] Westbury JL Gee P Ling T Brown DT Franks KH Bindoff I RedUSe: Reducing antipsychotic and benzodiazepine prescribing in residential aged care facilities. Med J Aust (2018) 208:398–403. 10.5694/mja17.00857 29747564

[45] Mao L Jin H Wang M Hu Y Chen S He Q Neurologic Manifestations of Hospitalized Patients with Coronavirus Disease 2019 in Wuhan, China. JAMA Neurol (2020) 77 (6):683–90. 10.1001/jamaneurol.2020.1127 PMC7149362 32275288

[46] Centers for Disease Control and Prevention Evaluating and Testing Persons for Coronavirus Disease 2019 (2020). Available at: https://www.cdc.gov/coronavirus/2019-ncov/hcp/clinical-criteria.html (Accessed May 20, 2020).

[47] American Society of Microbiology False Negatives and Reinfections: the Challenges of SARS-CoV-2 RT-PCR Testing (2020). Available at: https://asm.org/Articles/2020/April/False-Negatives-and-Reinfections-the-Challenges-of (Accessed May 20, 2020).

[48] Inouye SK van Dyck CH Alessi CA Balkin S Siegal AP Horwitz RI Clarifying Confusion: The Confusion Assessment Method. Ann Intern Med (1990) 113:941–8. 10.1002/9781444324617.ch29 2240918

[49] Infection Control Expert Group, Commonwealth of Australia. COVID-19 Infection Prevention and Control for Residential Care Facilities. Available at https://www.health.gov.au/resources/publications/coronavirus-covid-19-guidelines-for-infection-prevention-and-control-in-residential-care-facilities (Accessed May 20, 2020)

[50] Wang H Li T Barbarino P Gauthier S Brodaty H Molinuevo JL Dementia care during COVID-19. Lancet (2020) 395:1190–1. 10.1016/S0140-6736(20)30755-8 PMC7146671 32240625

[51] Agens JE Jr. Chemical and physical restraint use in the older person. Br J Med Pract (2010) 3:34–9.

[52] Machiels M Metzelthin SF Hamers JPH Zwakhalen SMG Interventions to improve communication between people with dementia and nursing staff during daily nursing care: A systematic review. Int J Nurs Stud (2017) 66:37–46. 10.1016/j.ijnurstu.2016.11.017 27951433

[53] Alzheimer’s Society of Canada Tips for Caregivers - Managing Through COVID-19 (2020). Available at: https://alzheimer.ca/en/Home/Living-with-dementia/managing-through-covid-19/tips-caregivers (Accessed May 20, 2020).

[54] Alzheimer’s Association Coronavirus (COVID-19): Tips for Dementia Caregivers (2020). Available at: https://alz.org/help-support/caregiving/coronavirus-(covid-19)-tips-for-dementia-care (Accessed June 2, 2020).

[55] Brodaty H Sc D Arasaratnam C Psych B Meta-Analysis of Nonpharmacological Interventions for Neuropsychiatric Symptoms of Dementia. Am J Psychiatry (2012) 169:946–53. 10.1176/appi.ajp.2012.11101529 22952073

[56] Mello M Persad G White D Respecting Disability Rights — Toward Improved Crisis Standards of Care. N Engl J Med (2020) 382:1978–9. 10.1056/NEJMp2009027 32427433

[57] Solomon M Wynia M Gostin L Covid-19 Crisis Triage — Optimizing Health Outcomes and Disability Rights. N Engl J Med (2020) 382:1978–9. 10.1056/NEJMp2009027 32427434

[58] Oczkowski SJ Chung HO Hanvey L Mbuagbaw L You JJ Communication tools for end-of-life decision-making in ambulatory care settings: A systematic review and meta-analysis. PloS One (2016) 11:1–21. 10.1371/journal.pone.0150671 PMC4847908 27119571

[59] Fotuhi M Mian A Meysami S Raji CA Neurobiology of COVID-19. J Alzheimer’s Dis (2020) 76:1–17. 10.3233/jad-200581 32538857 PMC7660990

[60] Filatov A Sharma P Hindi F Espinosa PS Neurological Complications of Coronavirus Disease (COVID-19): Encephalopathy. Cureus (2020) 12:1–6. 10.7759/cureus.7352 PMC7170017 32328364

[61] Varatharaj A Thomas N Ellul MA Davies NWS Pollak TA Tenorio EL Neurological and neuropsychiatric complications of COVID-19 in 153 patients: a UK-wide surveillance study. Lancet Psychiatry (2020) 2:1–8. 10.1016/s2215-0366(20)30287-x PMC7316461 32593341

[62] Paniz-Mondolfi A Bryce C Grimes Z Gordon RE Reidy J Lednicky J Central Nervous System Involvement by Severe Acute Respiratory Syndrome Coronavirus -2 (SARS-CoV-2). J Med Virol (2020) 2:0–3. 10.1002/jmv.25915 PMC7264598 32314810

[63] Politi LS Salsano E Grimaldi M Magnetic Resonance Imaging Alteration ofthe Brain in a Patient With Coronavirus Disease 2019 (COVID-19) and Anosmia. JAMA Neurol (2020) 92 (7):707–09. 10.1002/jmv.25824 32469400

[64] Merkler AE Parikh NS Mir S Gupta A Kamel H Lin E Risk of Ischemic Stroke in Patients With Coronavirus Disease 2019 (COVID-19) vs Patients With Influenza. JAMA Neurol (2020) 2019:1–7. 10.1001/jamaneurol.2020.2730 PMC7333175 32614385

[65] Ellul MA Benjamin L Singh B Lant S Michael BD Easton A Rapid Review Neurological associations of COVID-19. Lancet Glob Heal (2020) 4422:2–3. 10.1016/S1474-4422(20)30221-0 PMC7332267 32622375

[66] Kotfis K Williams Roberson S Wilson JE Dabrowski W Pun BT Ely EW COVID-19: ICU delirium management during SARS-CoV-2 pandemic. Crit Care (2020) 24:1–9. 10.1186/s13054-020-02882-x 32345343 PMC7186945

[67] Helms J Kremer S Merdji H Clere-Jehl R Schenck M Kummerlen C Neurologic Features in Severe SARS-CoV-2 Infection. N Engl J Med (2020) 382:2268–70. 10.1056/nejmc2008597 PMC7179967 32294339

[68] Pereira A Long-Term Neurological Threats of COVID-19: A Call to Update the Thinking About the Outcomes of the Coronavirus Pandemic. Front Neurol (2020) 11:308. 10.3389/fneur.2020.00308 32362868 PMC7182030

[69] Pollock BG Mulsant BH Between Scylla and Charybdis: antipsychotic and other psychotropic medications in older nursing home residents. CMAJ (2011) 183:778–9. 10.1503/cmaj.101406.Competing PMC3080524 21444612

[70] Vasudev A Shariff SZ Liu K Burhan AM Herrmann N Leonard S Trends in Psychotropic Dispensing among Older Adults with Dementia Living in Long-Term Care Facilities: 2004-2013. Am J Geriatr Psychiatry (2015) 23:1259–69. 10.1016/j.jagp.2015.07.001 26525997

[71] Livingston G Sommerlad A Orgeta V Costafreda SG Huntley J Ames D The Lancet International Commission on Dementia Prevention and Care. Lancet (2017) 390:2673–734. 10.1016/S0140-6736(17)31363-6 28735855

[72] Korczyn A Dementia in the Era of COVID-19 (International Behavioural Neurology Rounds). J Alzheimer's Dis (2020) 75(4):1071–2. 10.3233/JAD-200609 PMC7369036 32538858

[73] Long B Brady WJ Koyfman A Gottlieb M Cardiovascular complications in COVID-19. Am J Emerg Med (2020) 38(7):1504–7. 10.1016/j.ajem.2020.04.048 PMC7165109 32317203

[74] Kindermann SS Dolder CR Bailey A Katz IR Jeste DV Pharmacological treatment of psychosis and agitation in elderly patients with dementia: Four decades of experience. Drugs Aging (2002) 19:257–76. 10.2165/00002512-200219040-00002 12038878

[75] Troyer EA Kohn JN Hong S Are we facing a crashing wave of neuropsychiatric sequelae of COVID-19? Neuropsychiatric symptoms and potential immunologic mechanisms. Brain Behav Immun (2020) 87:34–9. 10.1016/j.bbi.2020.04.027 PMC7152874 32298803

[76] Vigo D Patten S Pajer K Krausz M Taylor S Rush B Mental Health of Communities during the COVID-19 Pandemic. Can J Psychiatry (2020) 1–7. 10.1177/0706743720926676 706743720926676. PMC7502878 32391720

[77] Brooks SK Webster RK Smith LE Woodland L Wessely S Greenberg N The psychological impact of quarantine and how to reduce it: rapid review of the evidence. Lancet (2020) 395:912–20. 10.1016/S0140-6736(20)30460-8 PMC7158942 32112714

[78] Mcmichael TM Clark S Pogosjans S Kay M Lewis J Baer A COVID-19 in a Long-Term Care Facility — King County, Washington. Morb Mortal Wkly Rep (2020) 69:339–42. 10.15585/mmwr.mm6912e1 PMC7725515 32214083

[79] Iaboni A Cockburn A Marcil M Rodrigues K Marshall C Garcia MA Achieving Safe, Effective, and Compassionate Quarantine or Isolation of Older Adults With Dementia in Nursing Homes. Am J Geriatr Psychiatry (2020) 28(8):835–8. 10.1016/j.jagp.2020.04.025 PMC7196899 32430111

[80] Davidson PM Szanton SL Nursing homes and COVID-19: we can and should do better. J Clin Nurs (2020) 29(15–16):2758–9. 10.1111/jocn.15297 PMC7262177 32281165

[81] Santini ZI Jose PE York Cornwell E Koyanagi A Nielsen L Hinrichsen C Social disconnectedness, perceived isolation, and symptoms of depression and anxiety among older Americans (NSHAP): a longitudinal mediation analysis. Lancet Public Heal (2020) 5:e62–70. 10.1016/S2468-2667(19)30230-0 31910981

[82] Scales K Zimmerman S Miller SJ Evidence-Based Nonpharmacological Practices to Address Behavioral and Psychological Symptoms of Dementia. Gerontologist (2018) 58:S88–S102. 10.1093/geront/gnx167 29361069 PMC5881760

[83] Lewis MM Trzinski AL Counseling older adults with dementia who are dealing with death: Innovative interventions for practitioners. Death Stud (2006) 30:777–87. 10.1080/07481180600853199 16972377

[84] Tan A How to talk to your loved ones & healthcare team about your wishes & goals if you become sick with COVID-19 (New Coronavirus) (2020). Available at: http://amytanmd.ucalgaryblogs.ca/files/2020/04/Guide-for-Talking-about-Wishes-Goals-with-COVID-19-Handout-by-Dr.-Amy-Tan-v3.pdf (Accessed May 20, 2020).

[85] Toot S Devine M Akporobaro A Orrell M Causes of Hospital Admission for People With Dementia: A Systematic Review and Meta-Analysis. J Am Med Dir Assoc (2013) 14:463–70. 10.1016/j.jamda.2013.01.011 23510826

[86] Tang WK Chiu H Woo J Hjelm M Hui E Telepsychiatry in psychogeriatric service: a pilot study. Int J Geriatr Psychiatry (2001) 16:88–93. 10.1002/1099-1166(200101)16:1<88::AID-GPS282>3.0.CO;2-W 11180491

[87] van der Ploeg ES Mbakile T Genovesi S OConnor DW The potential of volunteers to implement non-pharmacological interventions to reduce agitation associated with dementia in nursing home residents. Int Psychogeriatrics (2012) 24:1790–7. 10.1017/S1041610212000798 22613048

[88] Shultz JM Baingana F Neria Y The 2014 Ebola outbreak and mental health: Current status and recommended response. JAMA J Am Med Assoc (2015) 313:567–8. 10.1001/jama.2014.17934 25532102

[89] Lai J Ma S Wang Y Cai Z Hu J Wei N Factors Associated With Mental Health Outcomes Among Health Care Workers Exposed to Coronavirus Disease 2019. JAMA Netw Open (2020) 3:e203976. 10.1001/jamanetworkopen.2020.3976 32202646 PMC7090843

[90] Highfield J Johnston E Jones T Kinman G Maunder R Monaghan L The Psychological needs of healthcare staff as a result of the coronavirus pandemic. Br Psychol Soc (2020). Available online at: https://www.bps.org.uk/sites/www.bps.org.uk/files/News/News%20-%20Files/Psychological%20needs%20of%20healthcare%20staff.pdf

[91] Lancee WJ Maunder RG Goldbloom DS Prevalence of psychiatric disorders among Toronto hospital workers one to two years after the SARS outbreak. Psychiatr Serv (2008) 59:91–5. 10.1176/ps.2008.59.1.91 PMC2923654 18182545

[92] Minematsu A The Frequency of Family Visits Influences the Behavioral and Psychological Symptoms of Dementia (BPSD ) of Aged People. Psychol Sci (2006) 18(2):123–6. 10.1589/jpts.18.123

[93] Brodaty H Green A Koschera A Meta-Analysis of Psychosocial Interventions. J Am Geriatr Soc (2003) 51:657–64. 10.1034/j.1600-0579.2003.00210.x 12752841

